# Acute leaflet opening restriction caused by endarterectomy-like iliac artery intimal detachment entrapped within a self-expanding transcatheter aortic valve: a case report

**DOI:** 10.1093/ehjcr/ytag314

**Published:** 2026-05-04

**Authors:** Akihiko Sato, Yuuki Muto, Takeshi Shimizu, Takashi Igarashi, Yasuchika Takeishi

**Affiliations:** Department of Cardiovascular Medicine, Fukushima Medical University, 1 Hikarigaoka, Fukushima 960-1295, Japan; Department of Cardiovascular Medicine, Fukushima Medical University, 1 Hikarigaoka, Fukushima 960-1295, Japan; Department of Cardiovascular Medicine, Fukushima Medical University, 1 Hikarigaoka, Fukushima 960-1295, Japan; Department of Cardiovascular Surgery, Fukushima Medical University, 1 Hikarigaoka, Fukushima 960-1295, Japan; Department of Cardiovascular Medicine, Fukushima Medical University, 1 Hikarigaoka, Fukushima 960-1295, Japan

**Keywords:** TAVI, Transfemoral access, Vascular complication, Intimal detachment, Transcatheter heart valve dysfunction, Evolut, Case report

## Abstract

**Background:**

Acute transcatheter heart valve (THV) dysfunction immediately after transcatheter aortic valve implantation (TAVI) is uncommon but can be catastrophic. Mechanical obstruction by migrated vascular tissue is exceptionally rare.

**Case summary:**

An 80-year-old woman with symptomatic very severe aortic stenosis and high surgical risk underwent transfemoral TAVI with a 23-mm self-expanding Evolut FX valve (Medtronic, Minneapolis, MN, USA). Preprocedural computed tomography showed severe circumferential calcification of the left common iliac artery (CIA) with a preserved lumen (6.8 × 4.9 mm) and a small aortic annulus (area, 244.9 mm^2^; perimeter, 55.9 mm). After valve deployment, invasive haemodynamics showed no reduction in the transvalvular pressure gradient (mean/peak, 58/97 mmHg before vs. 63/98 mmHg after deployment). Transoesophageal echocardiography demonstrated a tubular structure restricting leaflet opening. Balloon post-dilatation resulted in transient echocardiographic improvement but was complicated by balloon rupture, and a mobile intravalvular structure persisted. Emergency surgical conversion was performed. Intraoperatively, a calcified tubular structure with a lumen-like appearance was found on the guidewire and was impinging on the THV leaflets. The THV was explanted, and surgical aortic valve replacement was performed. Histology confirmed calcified vascular intimal tissue, consistent with detachment from the left CIA. The postoperative course was uneventful, and the patient was discharged home.

**Discussion:**

In severely calcified iliofemoral access, endarterectomy-like intimal detachment can occur during device passage and, rarely, can migrate into a self-expanding THV, causing acute leaflet opening restriction. Early recognition using multimodality imaging and timely surgical bailout may be life-saving.

Learning points

- Endarterectomy-like iliac intimal detachment can occur during transfemoral transcatheter aortic valve implantation in severely calcified access vessels, even when large-bore sheath passage appears feasible.
- Persistently high invasive gradients with acute leaflet opening restriction on intraprocedural transoesophageal echocardiography should raise suspicion of mechanical obstruction by migrated tissue.
- If a mobile intravalvular structure persists and percutaneous resolution is uncertain, early heart-team discussion and timely surgical conversion may be the safest strategy.

## Introduction

Transfemoral transcatheter aortic valve implantation (TAVI) is an established treatment for severe symptomatic aortic stenosis, guided by heart-team decision-making and comprehensive preprocedural imaging.^1^ Although vascular access complications remain clinically relevant,^2,3^ acute transcatheter heart valve (THV) dysfunction immediately after valve deployment is rare yet may be catastrophic. We describe acute leaflet opening restriction caused by endarterectomy-like detachment of a calcified iliac intimal plaque that migrated into and became entrapped within a self-expanding THV, resulting in failed gradient reduction and emergent surgical conversion.

## Summary figure

**Figure:**
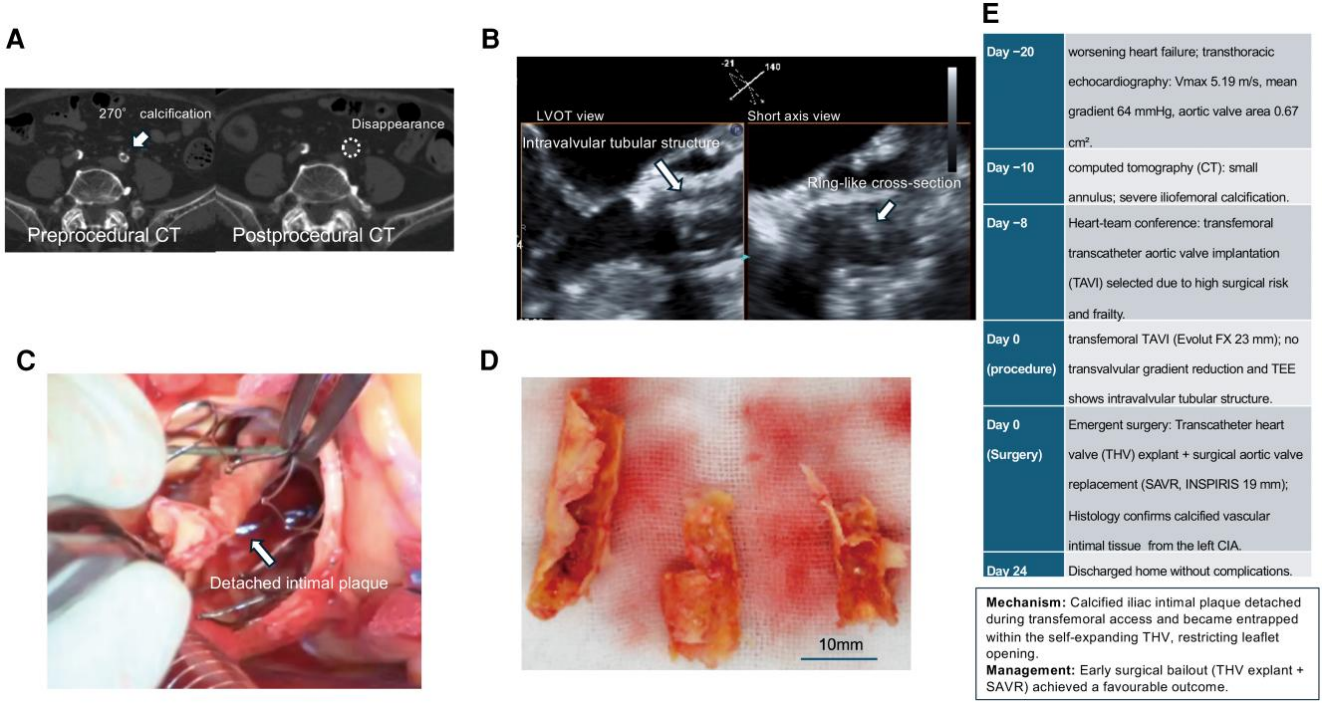
(*A*) Axial CT before and after the procedure showing a 270° circumferential calcified plaque in the patient’s left common iliac artery on preprocedural CT (arrow) and its disappearance on postprocedural CT (dotted circle), consistent with endarterectomy-like detachment. (*B*) Biplane long-axis transoesophageal echocardiography immediately after valve deployment demonstrating restricted leaflet opening due to an intravalvular tubular structure within the self-expanding transcatheter heart valve (arrow indicates the intravalvular tubular structure). (*C*) Intraoperative photograph showing a detached intimal plaque entrapped within the valve frame and mechanically impinging on a leaflet (arrow indicates the plaque). (*D*) Retrieved tubular calcified tissue consistent with vascular intimal origin (left common iliac artery). (*E*) Timeline of key clinical events.

## Case presentation

An 80-year-old woman with Type 2 diabetes mellitus and chronic kidney disease presented with worsening heart failure symptoms and was diagnosed with symptomatic very severe aortic stenosis. Physical examination revealed a systolic ejection murmur and mild peripheral oedema. Transthoracic echocardiography (TTE) showed a peak aortic jet velocity of 5.19 m/s, a mean transaortic gradient of 64 mmHg, and an aortic valve area of 0.67 cm^2^. Laboratory tests showed renal dysfunction (an estimated glomerular filtration rate of 23 mL/min/1.73 m^2^) and an elevated B-type natriuretic peptide level of 492.2 pg/mL. Given a high surgical risk (Society of Thoracic Surgeons score, 8.66%) and frailty (Clinical Frailty Scale, 4/9), the heart team selected TAVI in accordance with guideline-based practice.^1^

**Figure 1:**
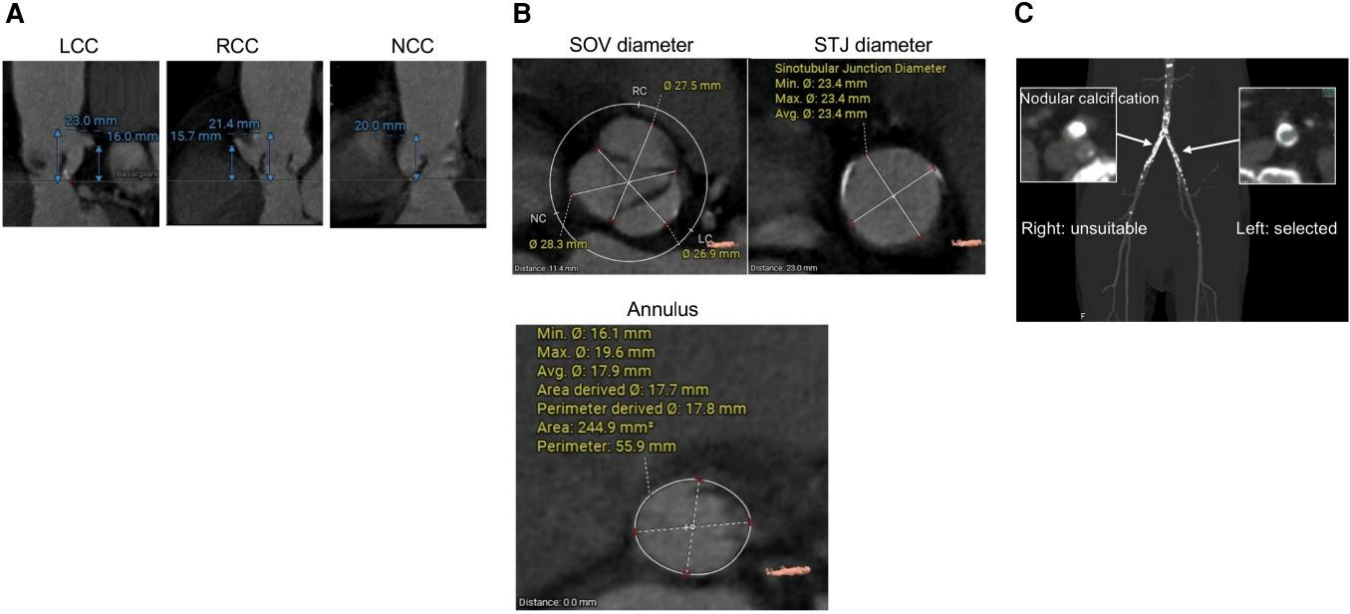
Preprocedural computed tomography assessment for procedural planning. (*A*) Coronary ostial heights from each cusp, including left coronary cusp (LCC), right coronary cusp (RCC), and non-coronary cusp (NCC), showing preserved coronary heights. (*B*) Computed tomography-derived measurements of the aortic valve complex used for transcatheter heart valve sizing, including annulus area/perimeter and dimensions of the sinuses of Valsalva and sinotubular junction. (*C*) Iliofemoral access assessment demonstrating severe iliac calcification: the right common iliac artery was unsuitable due to nodular calcification/stenosis, whereas the left side was selected for transfemoral access. The left common iliac artery lumen measured 6.8 × 4.9 mm.

Preprocedural contrast-enhanced computed tomography (CT) showed a tricuspid aortic valve with adequate coronary heights (*Figure 1A*). Computed tomography–derived aortic annular measurements were 244.9 mm^2^ in area and 55.9 mm in perimeter (*Figure 1B*). Iliofemoral access assessment revealed extensive calcification: the right common iliac artery (CIA) showed nodular calcification with stenosis and was deemed unsuitable, whereas the left CIA exhibited 270° circumferential calcification with a preserved lumen (6.8 × 4.9 mm) (*Figure 1C*). The circumferential calcification of the left CIA is shown on axial CT (*Summary figure*, panel *A*, left). A left transfemoral approach was planned.

**Figure 2:**
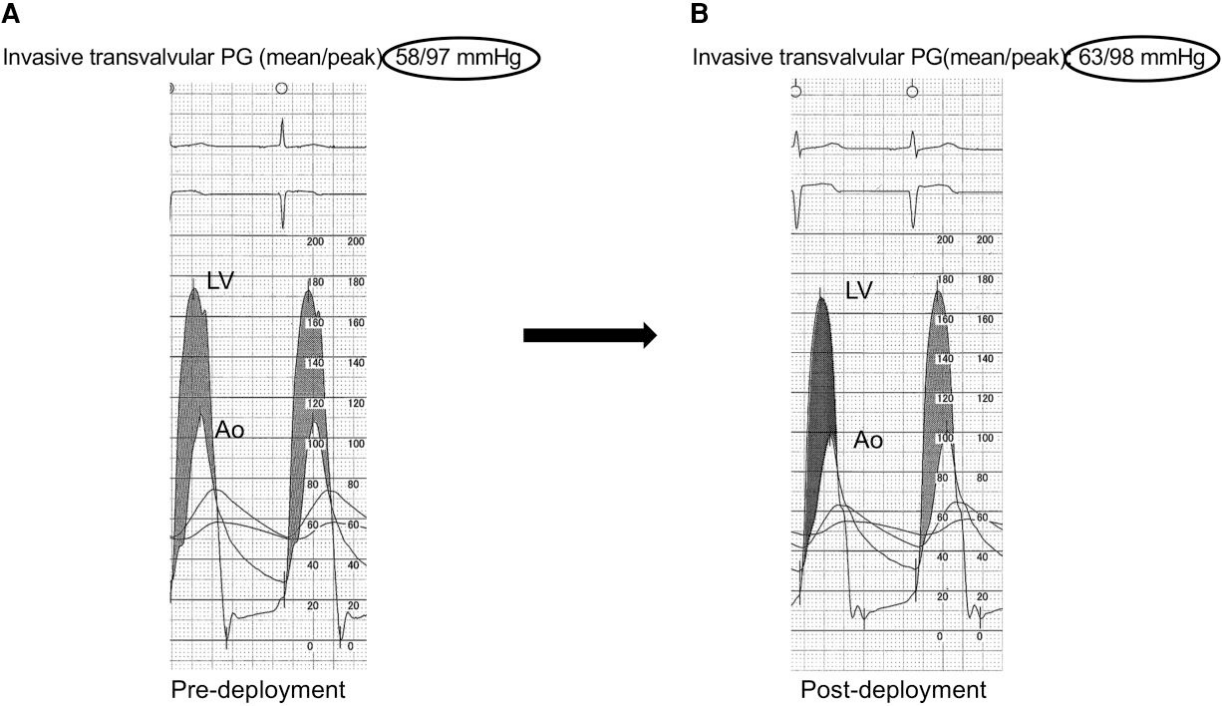
Invasive haemodynamic assessment demonstrating no reduction in transvalvular pressure gradient after transcatheter heart valve deployment. Simultaneous left ventricular–ascending aortic invasive pressure tracings show persistently high gradients (*A*) before and (*B*) after deployment (mean/peak pressure gradient 58/97 mmHg vs. 63/98 mmHg).

The procedure was performed under general anaesthesia. A 14-Fr GORE® DRYSEAL sheath (W. L. Gore & Associates, Flagstaff, AZ, USA) was advanced across the calcified left iliac segment to confirm crossability; if not feasible, direct aortic access was considered. Balloon aortic valvuloplasty was performed using an 18-mm INOUE BALLOON™ (Toray Industries, Inc., Tokyo, Japan). A 23-mm self-expanding Evolut FX valve (Medtronic, Minneapolis, MN, USA) was advanced; mild resistance was noted while crossing the left CIA. The valve was deployed using the cusp-overlap technique.^4^ Immediately after deployment, simultaneous left ventricular and ascending aortic (LV–Ao) pressure recordings showed no reduction in the invasive transvalvular pressure gradient (PG; mean/peak 58/97 mmHg pre-deployment vs. 63/98 mmHg post-deployment; *Figure 2*).

**Figure 3:**
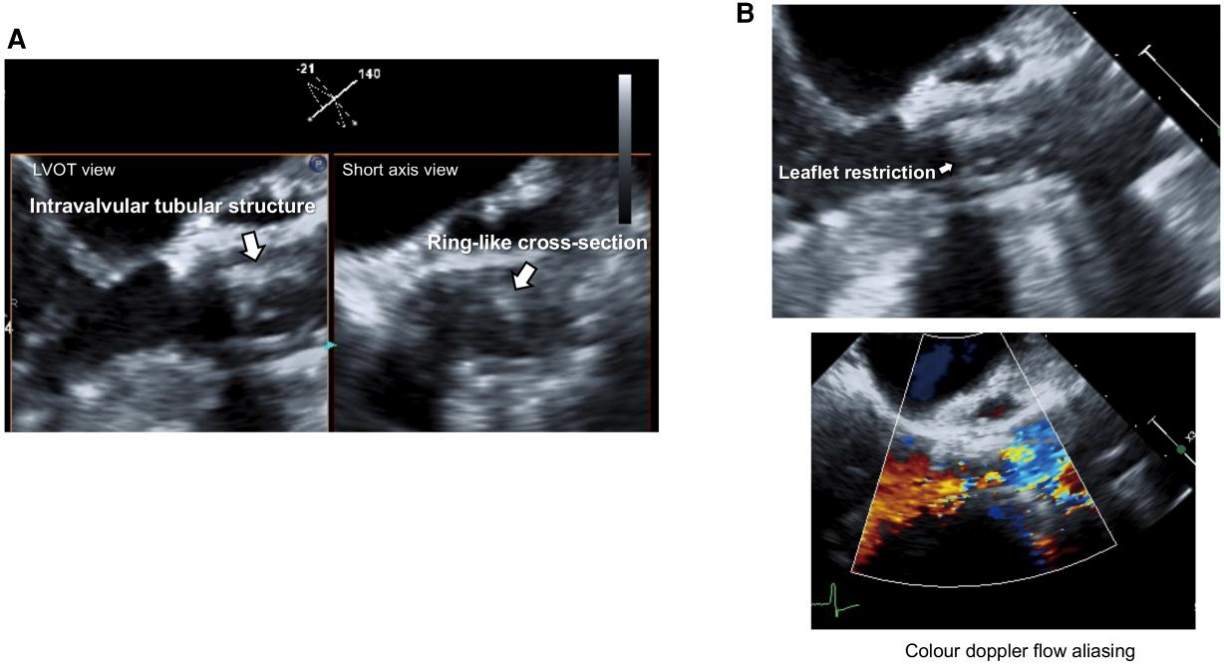
Transoesophageal echocardiography demonstrating acute leaflet opening restriction caused by an intravalvular tubular structure. (*A*) Biplane views showing an intravalvular tubular structure within the self-expanding transcatheter heart valve: tubular appearance in the left ventricular outflow tract (LVOT) view and ring-like in cross-section on the short-axis view (arrows indicate the intravalvular tubular structure). (*B*) Long-axis view demonstrating leaflet restriction (arrow) and colour Doppler showing flow aliasing across the transcatheter heart valve, consistent with significant functional obstruction.

Transoesophageal echocardiography (TEE) demonstrated restricted leaflet opening due to an intravalvular tubular structure within the THV (*Figure 3*).

Balloon post-dilatation using an 18-mm VACS-II balloon (Osypka AG, Rheinfelden, Germany) transiently improved leaflet motion but was complicated by balloon rupture, and a mobile intravalvular structure persisted.

Given persistent THV dysfunction and concern for embolization or recurrent obstruction if the structure was left *in situ*, emergent surgical conversion was undertaken.

In retrospect, fluoroscopy during device advancement showed cap-like adherent material migrating with the delivery system (*Video 1*); this was recognized only after surgical inspection.

The procedural sequence is summarized in the *Summary figure* (panel *E*).

**Figure 4:**
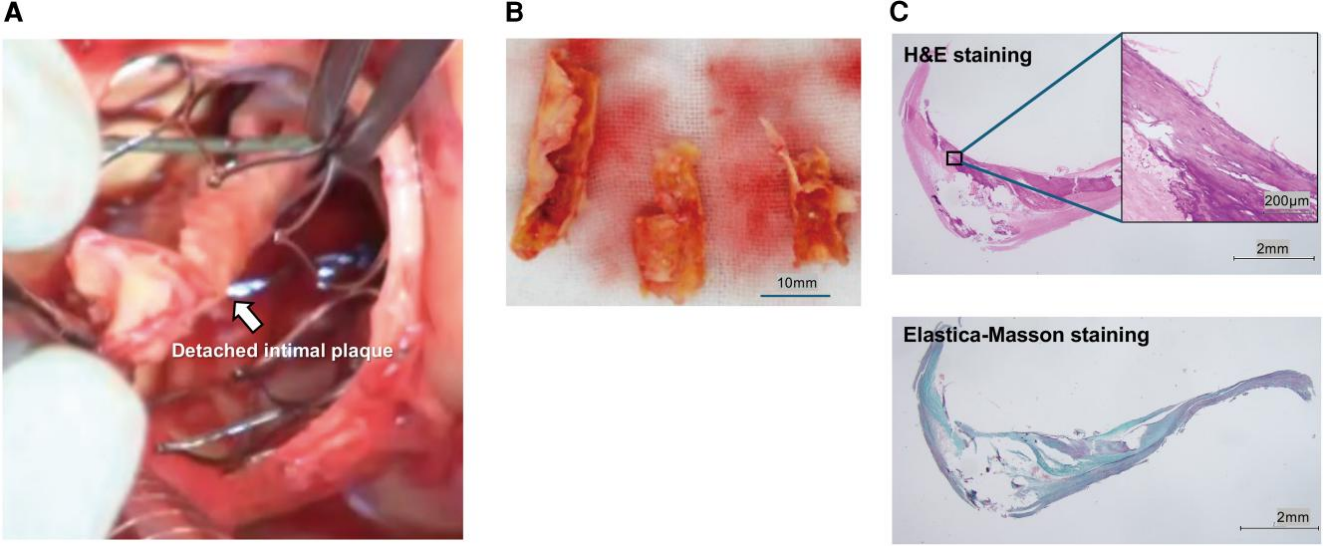
Surgical and pathological confirmation of intravalvular obstruction by a detached iliac intimal plaque. (*A*) Intraoperative view showing a calcified tubular structure with a lumen-like appearance within the self-expanding transcatheter heart valve frame, mechanically impinging on a transcatheter heart valve leaflet (arrow indicates the structure impinging on the leaflet). (*B*) Retrieved calcified tubular tissue consistent with endarterectomy-like intimal detachment (scale bars as shown). (*C*) Histological examination of the retrieved tissue. Upper panels: haematoxylin and eosin (*H&E*) staining demonstrating vascular intimal tissue with calcification and foamy macrophages (inset, higher magnification). Lower panel: Elastica–Masson staining highlighting the arterial wall structure, supporting an iliac arterial origin (scale bars as shown).

Intraoperatively, a calcified tubular structure with a lumen-like appearance was identified on the guidewire within the THV frame, mechanically impinging on a THV leaflet (*Figure 4A*). The Evolut valve was explanted, and surgical aortic valve replacement was performed using a 19-mm INSPIRIS bioprosthesis (Edwards Lifesciences Corporation, Irvine, CA, USA). Histological examination of the retrieved structure confirmed calcified vascular intimal tissue, consistent with detachment from the left CIA (*Figure 4B* and *C*). Postoperative TTE demonstrated good function of the surgical bioprosthesis. Final iliofemoral angiography showed no evidence of rupture or perforation, with only a mild iliac dissection and preserved distal flow. The postoperative course was uneventful, and the patient was discharged home on Day 24. Follow-up CT at a similar level showed disappearance of the calcified plaque at the implicated left iliac segment (*Summary figure*, panel *A*, right).

## Discussion

This case illustrates a rare mechanism of acute THV dysfunction: endarterectomy-like detachment of a calcified iliac intimal plaque during transfemoral access, which migrated into a self-expanding THV and caused acute leaflet opening restriction. A key diagnostic clue was the discordance between apparently successful valve deployment and persistently severe haemodynamics, documented by LV–Ao pressure recordings, prompting immediate multimodality reassessment.

When transvalvular gradients fail to improve immediately after THV deployment, common considerations include malposition, severe underexpansion, acute coronary obstruction with haemodynamic compromise, or—less commonly—leaflet thrombosis. In the present case, post-deployment fluoroscopy showed good stent-frame expansion without features suggestive of severe underexpansion. Moreover, TEE directly demonstrated an intravalvular tubular structure mechanically impinging on the leaflets (*Figure 3*). Together, these findings supported mechanical obstruction rather than underexpansion as the primary mechanism. Echocardiography is pivotal for the intraprocedural assessment of THV function and complications,^5^ and in this case, it enabled rapid identification of the obstructive structure.

Balloon post-dilatation with an 18-mm VACS-II resulted in transient improvement in leaflet opening on TEE, suggesting that the obstruction was at least partially deformable. Balloon expansion may have partially fractured or compressed the calcified plaque, allowing improved leaflet excursion; conversely, interaction with the calcified structure likely contributed to balloon rupture. Persistence of a mobile intravalvular structure after post-dilatation represented an unacceptable risk of embolization or recurrent obstruction, prompting early surgical bailout.

We propose the following mechanism. A circumferential iliac intimal plaque may have been inadvertently detached during transfemoral device passage and migrated cephalad along the delivery system and/or guidewire. After THV deployment, catheter manipulation over the wire for post-deployment assessment likely advanced the tubular plaque into the THV frame, where it became pinned against a leaflet, resulting in acute mechanical leaflet opening restriction and persistent invasive gradients. This sequence was recognized only after surgical conversion and retrospective review.

A mechanistically similar phenomenon has been reported by Reuthebuch *et al*., who described intimal disruption of the left subclavian artery during transsubclavian TAVI, with a highly mobile tubular ‘intimal cylinder’ migrating into the prosthesis and requiring surgical extraction; in that report, the access vessel injury subsequently manifested as subclavian dissection/stenosis, while prosthesis function was preserved at discharge.^6^ In contrast, our case involved iliofemoral left CIA endarterectomy-like detachment during transfemoral TAVI, with immediate functional valve malfunction (no gradient reduction; *Figure 2*) due to plaque entrapment and leaflet opening restriction (*Figure 3*), necessitating emergent surgical conversion. Thus, migrated intimal tissue can present not only as an access-site complication but also—rarely—as acute THV dysfunction.

Vascular complications after transfemoral TAVI remain clinically important,^3,7^ and although transfemoral access can be feasible even in heavily calcified vessels,^8^ this case highlights that endarterectomy-like intimal detachment may occur despite apparently acceptable lumen dimensions and may present as acute THV dysfunction rather than overt access-site complications. Therefore, preprocedural CT-based access planning should consider not only minimal lumen calibre but also the circumferential extent/length and depth of calcification, as well as vessel tortuosity, which may increase device passage difficulty and the risk of intimal injury. In our case, transfemoral access was selected because the calcified segment was short and non-tortuous and crossability was confirmed with a 14-Fr DRYSEAL sheath; if not feasible, direct aortic access was considered. At the time in our institution, transcarotid access was not available, whereas trans-subclavian and direct aortic approaches were established alternatives, and we planned to proceed with direct aortic access if transfemoral passage was not feasible. We also note a limitation of a ‘sheath-first’ strategy: the 14-Fr DRYSEAL sheath has excellent trackability, and successful sheath passage does not necessarily guarantee atraumatic passage of the subsequent valve delivery system. Prompt recognition using invasive haemodynamics, TEE, and fluoroscopy (*Video 1*), together with decisive heart-team management—including early surgical conversion when percutaneous resolution is not achievable—may be life-saving. Notably, completion iliofemoral angiography showed no rupture or perforation and only mild dissection, underscoring that clinically significant intimal detachment can occur even in the absence of overt angiographic catastrophe.

## Conclusion

Endarterectomy-like intimal detachment of the iliac artery can complicate transfemoral TAVI, and detached tissue can rarely migrate into a self-expanding THV, causing acute leaflet opening restriction. Prompt recognition using TEE and fluoroscopy and timely surgical conversion resulted in a favourable outcome.

## Data Availability

The data underlying this article will be shared on reasonable request to the corresponding author, in accordance with patient confidentiality and institutional requirements.

## Lead author biography

Dr Akihiko Sato graduated from Fukushima Medical University in 2009 and completed his doctoral programme at the same institution. He is currently based at Fukushima Medical University Hospital and specializes in catheter-based interventions for ischaemic heart disease and structural heart disease.

## Notes

**Consent:** Written informed consent was obtained from the patient for publication of this case report and accompanying images.
